# Strategies for HCC target discovery

**DOI:** 10.18632/aging.101233

**Published:** 2017-04-28

**Authors:** Pedro Molina-Sánchez, Amaia Lujambio

**Affiliations:** Department of Oncological Sciences, Icahn School of Medicine at Mount Sinai, New York, NY 10029, USA

**Keywords:** hepatocellular carcinoma, precision medicine, loss-of-function screens, RNAi, CRISPR

Liver cancer is now considered the sixth most prevalent malignancy and the second leading cause of cancer-related mortality in the world. In particular, hepatocellular carcinoma (HCC) is the most frequent liver cancer subtype, representing around 90% of all cases [1]. Unlike the majority of tumor types, it is expected that HCC incidence will increase drastically in the coming years, in part due to the lack of effective therapies [1]. To date, the multikinase inhibitor sorafenib is the only FDA-approved molecule for advanced HCC patients but unfortunately confers a limited survival benefit of 2-3 months on average [1]. One of the main challenges that HCC treatment faces is the high genetic heterogeneity among patients, which is largely attributed to the diversity of risk factors associated with its development (e.g. cirrhosis, hepatitis virus infection, alcohol abuse, or metabolic syndrome). Moreover, each malignant HCC nodule carries an average of 40 somatic mutations, making each tumor highly complex and unique [1]. This vast genetic variability among HCC patients indicates that patient-specific therapeutic strategies will be needed, further supporting the implementation of personalized therapies in HCC.

The development of new drugs is an arduous task that starts with the identification of the candidate targets. In principle, the ideal cancer target should only impact the survival and fitness of the cancer cells but spare the normal ones. One option is to focus the efforts on targeting cancer driver genes, which actively promote cancer progression and whose inhibition can lead to tumor reduction. Since many known cancer drivers are in practice undruggable, one alternative is to aim at targeting key mediators of those driver genes, as their repression should in theory render similar effects. Another possibility is to inhibit synthetic lethal targets, a strategy that is only effective in the presence of a particular genetic alteration present in the cancer cells [2]. To systematically identify novel drug targets we can now take advantage of robust genetic tools that allow the systematic analysis of a large number of potential genetic contributors. Thus, loss-of-function approaches, such as RNAi (RNA interference) and CRISPR screens, can be harnessed to identify genes that are essential for cellular proliferation and viability in a given genetic configuration [3], and therefore, putative genotype-specific anticancer targets.

RNAi screens have been used for this purpose during the last decade with satisfactory results. Unlike “genome-wide” screens, which are usually tedious, expensive, and hard to analyze and reproduce, “targeted” screens, focused on specific pathways or cellular processes, have emerged as a more convenient alternative for drug target discovery in cancer. One of the earliest studies successfully exploiting this concept was performed in acute myeloid leukemia. By using a customized short hairpin RNA (shRNA) library targeting 243 known chromatin regulators, BRD4 (Bromodomain-containing protein 4) was identified as a promising drug target [4], a finding that has already been translated into clinical trials. In HCC, a similar strategy was followed by employing an shRNA library designed against available drug target genes. This study identified CDK9 (Cyclin dependent kinase 9) as a drug target for those tumors expressing high levels of MYC, which is an undruggable driver in HCC, [5], providing, not only a novel therapeutic strategy, but also a patient selection criteria.

More recently, CRISPR technology has been adapted for its use in high-throughput genetic screenings. Pioneering works have confirmed the high sensitivity of CRISPR-based libraries to identify essential genes in human cancer cell lines [6], highlighting the potential of this approach to reveal cancer-related vulnerabilities. While it is too early to clinically translate the drug targets identified through CRISPR screens, additional studies have identified novel therapeutic targets in a variety of tumor types, further confirming the possibilities of this new technology. In HCC and by following a different strategy, an *in vivo* CRISPR screen unmasked four novel tumor suppressor genes (NF1, PLXNB1, FLRT2, and B9D1) that are negative regulators of the MAP kinase pathway, indicating that drugs targeting the pathway could be successful in treating HCC patients harboring mutations in any of the four genes [7].

Despite the very promising prospects that these technologies exhibit, there are still some obstacles that may hinder the identification of patient-specific targets in HCC. On one hand, both types of technologies present some limitations. For instance, RNAi leads to incomplete target inhibition and produces significant off-target effects, contributing to false negative and false positive results, respectively. Furthermore, while CRISPR technology seems to overcome these drawbacks, it also induces double strain breaks in the DNA, which trigger genotoxic stress and limit its use for targeting highly amplified loci (very frequent in cancer cells) [3]. In this regard, recent studies suggest that the combination of both technologies could reduce most of these issues [3]. On the other hand, the molecular and genetic bases of HCC are complex and variable, limiting the faithfulness of the available models. Taken together, loss-of-function tools combined with accurate models of human HCC have the potential to significantly broaden the therapeutic options for HCC patients and facilitate the implementation of precision medicine for this deadly disease.

## References

[1] Llovet JM (2016). Nat Rev Dis Primers.

[2] Manchado E (2017). Cold Spring Harb Symp Quant Biol.

[3] Housden BE (2017). Nat Rev Genet.

[4] Zuber J (2011). Nature.

[5] Huang CH (2014). Genes Dev.

[6] Wang T (2015). Science.

[7] Song CQ (2017). Gastroenterology.

